# UDFF and Auto pSWE accurately assess liver steatosis and fibrosis risk in obese patients with MASLD

**DOI:** 10.1055/a-2592-1431

**Published:** 2025-08-07

**Authors:** Nina Dominik, Larissa Nixdorf, Michael Schwarz, Benedikt Silvester Hofer, Lukas Hartl, Lorenz Balcar, Georg Semmler, Benedikt Simbrunner, Laurenz Fritz, Laurenz Hauptmann, Mathias Jachs, Julia Jedamzik, Behrang Mozayani, Lisa Gensthaler, Daniel Moritz Felsenreich, Mattias Mandorfer, Felix Langer, Michael Trauner, Thomas Reiberger, Gerhard Prager, David JM Bauer

**Affiliations:** 1 Internal Medicine III, Division of Gastroenterology and Hepatology, Medical University of Vienna, Vienna, Austria; 2 27271 Vienna Hepatic Hemodynamic Lab, Medical University of Vienna, Vienna, Austria; 3 Division of General Surgery and Metabolic- and Bariatric Surgery, Department of Surgery, Medical University of Vienna, Vienna, Austria; 4 Division of Cardiology, Department of Medicine II, Medical University of Vienna, Vienna, Austria; 5 Department of Pathology, Medical University of Vienna, Vienna, Austria; 6 Division of Gastroenterology and Hepatology, Klinik Ottakring, Vienna, Austria

**Keywords:** Point of care, Education, Training, Quality Assurance, MASLD, Auto pSWE, UDFF

## Abstract

**Background:**

Metabolic dysfunction-associated steatotic liver disease (MASLD) can progress to fibrosis and cirrhosis. Fibrosis and steatosis assessment with vibration-controlled transient elastography (VCTE) and controlled attenuation parameter (CAP) requires a dedicated device and time to obtain ≥10 reliable measurements. Auto pSWE allows for the simultaneous collection of 15 ARFI-based liver stiffness measurements (LSM) and UDFF-based steatosis assessment in a single acquisition.

**Methods:**

This prospective study included patients undergoing liver biopsy, primarily during bariatric surgery, between 11/2021–12/2023. Paired LSM by Auto pSWE/VCTE and steatosis assessments by UDFF/CAP were performed within a median of 1 day before or after biopsy.

**Results:**

134 patients (65% women, mean age: 42.6±13.3 years) with a high prevalence of obesity (mean BMI: 42.7±10.4; MASLD etiology: 88%) were included. Liver biopsy showed significant fibrosis (≥F2) in 27% of patients and moderate steatosis (≥S2) in 51%. A single 1×15 Auto pSWE acquisition and one UDFF measurement were as accurate as the median of 5 measurements. Auto pSWE (AUC: ≥F2=0.58, ≥F3=0.96, F4=0.97) and VCTE (AUC: ≥F2=0.60, ≥F3=0.92, F4=0.93) demonstrated high accuracy for advanced fibrosis stages. UDFF (AUC: ≥S1=0.79, ≥S2=0.78, S3=0.67) and CAP showed similar diagnostic accuracy.

**Conclusion:**

Auto pSWE and UDFF provide accurate, noninvasive tests for advanced liver fibrosis and steatosis in MASLD, even in severely obese patients. Notably, Auto pSWE captures 15 LSM with UDFF in a single acquisition, saving time and eliminating the need for a dedicated device.

## Zusammenfassung

**Hintergrund:**

Die mit Stoffwechselstörungen assoziierte steatotische Lebererkrankung (Metabolic dysfunction-associated steatotic liver disease; MASLD) kann zu Fibrose und Zirrhose führen. Die nicht-invasive Beurteilung von Fibrose und Steatose mittels vibrationskontrollierter transienter Elastografie (VCTE) und Controlled-Attenuation-Parameter-(CAP-)Technologie erfordert ein spezielles Gerät und mindestens 10 verlässliche Messungen. Die ultraschall-basierte Fettfraktion (UDFF) und die Auto-Punkt-Scherwelle (Auto-pSWE) ermöglichen eine Quantifizierung von Lebersteatose und -fibrose in nur einer Akquisition.

**Methoden:**

Diese prospektive Studie umfasste Patient:innen, die zwischen 11/2021 und 12/2023 eine Leberbiopsie erhielten. Paarweise Lebersteifigkeits- und Steatosemessungen durch Auto pSWE/UDFF und VCTE/CAP wurden einen Tag vor oder nach der Leberbiopsie durchgeführt.

**Ergebnisse:**

134 Patient:innen mit hoher Adipositas-Prävalenz wurden eingeschlossen. Eine einzige Akquisition (welche 15 pSWE-Messungen erfasst) durch Auto-pSWE lieferte dieselbe diagnostische Genauigkeit wie der Median aus 5 Messungen. Auto-pSWE (≥F2, AUC=0,58; ≥F3, AUC=0,96; F4, AUC=0,97) und VCTE zeigten eine hohe diagnostische Genauigkeit für die Erfassung einer fortgeschrittenen Fibrose. Die Diagnosegenauigkeit von UDFF (≥S1, AUC=0,79; ≥S2, AUC=0,78) war vergleichbar mit CAP.

**Schlussfolgerung:**

Auto pSWE und UDFF sind präzise, nicht-invasive Methoden zur Diagnostik fortgeschrittener Leberfibrose und -steatose bei Patient:innen mit MASLD. Die simultane Erfassung von 15 pSWE-Messungen und UDFF in einer Akquisition ist zeitsparend und erfordert kein spezielles Gerät.

## Introduction

The prevalence of metabolic dysfunction-associated steatotic liver disease (MASLD) is rapidly increasing 1 . Without intervention, MASLD can advance to liver cirrhosis and cause considerable morbidity and mortality 2 . Ultrasound-based assessment of MASLD focuses on steatosis and fibrosis risk, indicating metabolic burden and risk of liver-related events. Steatosis can be assessed visually or via controlled attenuation parameter (CAP) on vibration-controlled transient elastography (VCTE) 3 , though both lack diagnostic accuracy 4 . Recently, ultrasound-derived fat fraction (UDFF) has become available and represents a promising tool for the noninvasive assessment of liver steatosis and its quantification, similar to proton density fat fraction (PDFF) on magnetic resonance imaging (MRI) 5 . UDFF’s diagnostic accuracy and optimal cut-off values have not yet been sufficiently established.

Liver fibrosis risk can be routinely evaluated noninvasively using VCTE, point shear wave (pSWE), and two-dimensional shear wave (2D-SWE) technologies. In patients with obesity, high-powered transducers, or in case of VCTE, the XL-probe may be used to improve accuracy and success rates 3 6 7 . More recently, auto point shear wave elastography (Auto pSWE), a modality that measures 12–15 liver stiffness measurements (LSMs) in a single acquisition, promises to reduce acquisition times and improve accuracy 8 . These potential advantages of Auto pSWE have yet to be evaluated.

Our prospective, biopsy-controlled study aimed to evaluate (i) the diagnostic accuracy of UDFF and CAP for risk assessment of steatosis using liver biopsy as the reference standard and (ii) the diagnostic accuracy of Auto pSWE and VCTE for risk assessment of liver fibrosis using liver biopsy as the reference standard.

## Patients and Methods

### Study Cohort

This prospective, single-center study, conducted between 11/2021 and 12/2023, included patients with severe obesity scheduled for metabolic/bariatric surgery and those with suspected or diagnosed steatotic liver disease, attending the outpatient clinic or scheduled for liver biopsy at the Vienna General Hospital (AKH)/Medical University of Vienna. UDFF and Auto pSWE were performed within a median of 1 day (IQR: 1–2) before or after biopsy. The study excluded individuals with suspected or confirmed porto-sinusoidal vascular disease (PSVD), liver-related tumor, liver-related thrombosis including portal vein thrombosis, mechanical cholestasis or a transaminase flare (defined as alanine transaminase > 5× upper normal limit), due to their impact on SWE and ultrasound-based liver steatosis assessment. Informed consent was obtained from all participants.

### Elastography Protocol

Liver stiffness and steatosis were assessed using VCTE-LSM, CAP, Auto pSWE and UDFF. VCTE-LSM and CAP were conducted using FibroScan Expert 630 (Echosens, Paris, France) while Auto pSWE and UDFF were performed using the DAX transducer on the Siemens ACUSON Sequoia system (Siemens Healthineers Ultrasound, Issaquah, Washington, USA; Software Revision VA30). Six operators DB, ND, GS, LB, LH, MS, each with a track record of over 400 elastography procedures, conducted the assessments, supervised by DB & TR. Participants fasted for four hours prior to the procedures 9 and were positioned supine with their right arm elevated. Measurements employed an intercostal approach along the midaxillary line. For VCTE-LSM, the M-probe was used initially, transitioning to the XL-probe as advised by the probe selection tool 10 . Ten successful measurements were recorded per participant, with reliability defined by IQR/Med ≤30% or LSM <7.1 kPa 11 .

For Auto pSWE-LSM, the ROI was positioned to avoid interference from ribs and vessels. Measurements were taken at least 2 cm beneath the liver capsule, with at least 10 VCTE-LSM and 5 Auto pSWE-LSMs per patient. Reliability for Auto pSWE was defined by IQR/Med ≤30% 9 . In addition to LSM, 10 CAP measurements were taken, with the median value calculated for analysis. Continuous CAP (cCAP) was measured until the device indicated completion. For UDFF, 5 measurements were acquired and the median was calculated. Notably, no specific quality criteria were applied for liver steatosis assessment. Failed measurements were defined as the inability to achieve the required number of measurements within 30 attempts. Auto pSWE/VCTE and UDFF/CAP measurements were conducted before or after liver biopsy, with 45 patients assessed prior to biopsy and 89 after biopsy.

### Liver biopsy

Patients undergoing laparoscopic metabolic/bariatric surgery received liver biopsies using a direct-vision needle biopsy technique, focusing on the left hepatic lobe. Percutaneous liver biopsies were focused on the right hepatic lobe. The biopsy yielded liver core samples typically measuring 10 to 15 millimeters. Transjugular liver biopsies were obtained during the clinical procedure of hepatic venous pressure gradient (HVPG) measurement 12 . For histological analysis, the NAFLD activity score was applied to MASLD cases. For other conditions, pathologists utilized appropriate etiology-specific scoring systems based on the diagnosis.

### Laboratory and clinical parameters

Blood samples, including the enhanced liver fibrosis (ELF) test (Siemens Healthineers Diagnostics, New York, USA), were analyzed in the hospital's ISO-15189-certified laboratory. Clinical data were collected from direct measurements, patient histories, and electronic records, encompassing biometric data and diagnoses. Laboratory values are expressed as a percentage of the upper limit of normal (ULN). ULN values followed standard guidelines with separate references for men and women 13 . Median and interquartile ranges (IQR) are reported.

### Statistical analysis

Categorical variables were reported as absolute (n) and relative frequencies (%), with group comparisons using the Chi-squared test. Normally distributed variables were presented as mean ± standard deviation (SD), and non-normally distributed variables as median [IQR], with normality assessed by biological rationale or the Shapiro-Wilk test. Correlations were analyzed using Spearman coefficients, and beeswarm/violin plots visualized the median values. The diagnostic accuracy of Auto pSWE/VCTE for liver fibrosis as well as UDFF/CAP for liver steatosis was determined by calculating the receiver operator characteristics (ROC) and deriving the area under the ROC curve (AUC). We compared the AUC of the first measurement to the median of five measurements for each steatosis grade or fibrosis stage, using 2000 bootstrap iterations to assess differences. Correlation strengths and AUCs were interpreted following established guidelines 14 15 . Cut-offs for significant (≥F2) and advanced (≥F3) fibrosis, and mild (≥S1), moderate (≥S2), and severe (≥S3) steatosis were determined using the Youden Index and sensitivity/specificity aims (90% specificity for rule-in, 90% sensitivity for rule-out). Analyses were conducted in R (version 4.3.3), with detailed methods and packages provided in the Supplementary Methods.

## Results

### Patient characteristics

**Fig. 1:**
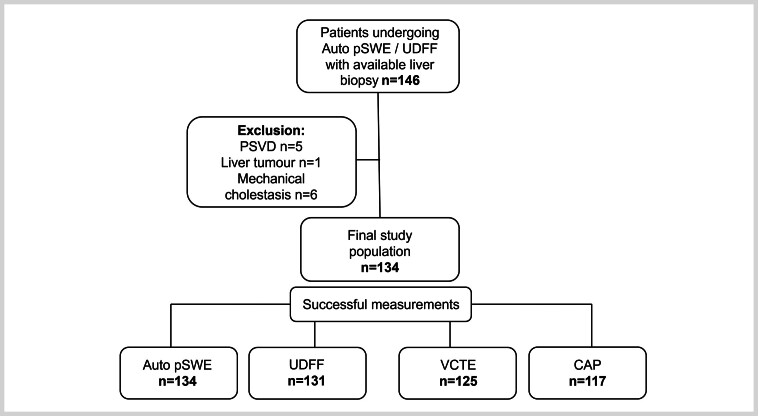
Patient flowchart. Abbreviations: Auto pSWE: automated point shear wave elastography; CAP: controlled attenuation parameter; DAX: deep abdominal (transducer); PSVD: porto-sinusoidal vascular disorder; UDFF: ultrasound-derived fat fraction; VCTE: vibration-controlled transient elastography

In this prospective study, 146 patients with liver biopsy underwent paired LSM (Auto pSWE and VCTE) and steatosis assessment (UDFF and CAP). After applying the exclusion criteria, 134 patients were included ( Fig. 1 ), including 97 patients undergoing liver biopsy during metabolic/bariatric surgery, 33 via percutaneous liver biopsy, and 4 via transjugular liver biopsy at the Vienna Hepatic Hemodynamic Lab at Medical University of Vienna. Of these 134 patients, 60 were also part of a previous study 15 , which assessed the discriminative ability of two distinct elastography techniques, pSWE and 2D-SWE, for fibrosis staging, particularly using the DAX transducer. The current study expands the scope by assessing both fibrosis and steatosis quantification, with liver histology as the reference standard. Specifically, we evaluate the diagnostic performance of the novel Auto pSWE technique for fibrosis staging, as well as the novel UDFF technique for steatosis quantification. Finally, this study explores the combined utility of these techniques for potential implementation in clinical practice.

Participants had a median age of 42.6±13.3 years, 65% were female, and the mean BMI (body mass index) was 42.7±10.4 kg/m ^2^ . The most prevalent etiology of liver disease was MASLD (n=118, 88%). The median VCTE and Auto pSWE values were 6.6 [IQR: 5.3] kPa and 3.1 [IQR: 1.6], respectively ( Table 1 ). The fibrosis stages on liver biopsy were: F0, n= 83 (62%); F1, n=14 (10%); F2, n=28 (21%); F3, n=2 (2%); and F4, n=7 (5%; **Supplementary Table 1** ). Patient characteristics stratified by fibrosis stage are provided in **Supplementary Table 2** . Biopsy quality parameters are summarized in **Supplementary Table 3** . The median CAP was 292 [IQR: 60] dB/m and the median UDFF was 18 [IQR: 16] %. Reflecting this, steatosis grades on liver biopsy were: S0, n=25 (19%); S1, n=38 (28%); S2, n=41 (31%); S3, n=30 (22%; **Supplementary Table 4** ). Some patients underwent cCAP examinations, showing no significant difference compared to conventional CAP (median CAP 295 [IQR: 78] vs. cCAP 300 [IQR: 69] dB/m, p=0.608), with similar correlations to histologic steatosis ( **Supplementary Table 5** ). Successful and reliable measurements were achieved in 125 (95.4%) for VCTE-LSM, 134 (100%) for Auto pSWE-LSM, 117 (87.3%) for CAP, and 131 (97.7%) for UDFF ( Fig. 1 ). Further details on liver biopsy results by fibrosis and steatosis grades are presented in **Supplementary Tables 1 and 2** .

**Table:** **Table 1** Patient characteristics of the overall cohort.

Patient characteristics	Overall, n=134
Age, years, mean ± SD	42.6 ± 13.3
Sex, n (%)	
Male	47 (35.1%)
Female	87 (64.9%)
BMI, kg/m ^2^ , mean ± SD	42.7 ± 10.4
BMI >30 kg/m2, 118 (88%)	
BMI >40 kg/m2, 90 (67%)	
Platelets, G/L, median [IQR]	249 [106]
INR (% of ULN), median [IQR]	6 [17]
Bilirubin (% of ULN), median [IQR]	157 [106]
Albumin, g/dL, median [IQR]	40.9 [5.1]
MELD-Na, points, median [IQR]	7 [2]
ALT (% of ULN), median [IQR]	94 [78]
AST (% of ULN), median [IQR]	69 [35]
ALP (% of ULN), median [IQR]	65 [28]
GGT (% of ULN), median [IQR]	160 [75]
HbA1c, %, median [IQR]	5.5 [1.4]
ELF test, pts, median [IQR]	8.9 [1.9]
Etiology, n (%)	
MASLD	118 (88.1%)
ArLD	3 (2.2%)
Viral	2 (1.5%)
Others	11 (8.2%)
VCTE probe, n (%)	
M	35 (26.1%)
XL	92 (68.7%)
Failed	7 (5.2%)
VCTE, kPa, median [IQR]	6.6 [5.3]
VCTE examination time, minutes, median [IQR]	4min 40s
Auto pSWE – median of 5 composite acquisitions, kPa, median [IQR]	3.1 [1.6]
Auto pSWE, time for 5 composite acquisitions, median [IQR]	50s
Auto pSWE – single composite acquisition, kPa, median [IQR]	3.0 [2.0]
Auto pSWE, time for single composite acquisitions, median [IQR]	8s
UDFF median of 5 acquisitions, %, median [IQR]	18 [16]
UDFF median – single composite acquisition, %, median [IQR]	18 [14]
CAP, dB/m, median [IQR]	292 [60]
Abbreviations: ALT: alanine aminotransferase; ALP: alkaline phosphatase; ArLD: alcohol-related liver disease; AST: aspartate aminotransferase; Auto pSWE: automated point shear wave elastography; BMI: body mass index; CAP: controlled attenuation parameter; DAX: deep abdominal (transducer); ELF: enhanced liver fibrosis test; GGT: gamma-glutamyl transferase; HbA1c: glycated hemoglobin; INR: international normalized ratio; IQR: interquartile range; MASLD: metabolic dysfunction-associated liver disease; MELD-Na: model for end-stage liver disease sodium score; n: number; SD: standard deviation; UDFF: ultrasound-derived fat fraction; ULN: upper limit of normal; VCTE: vibration-controlled transient elastography.	

### LSM by Auto pSWE vs. by VCTE for noninvasive fibrosis assessment

**Fig. 2:**
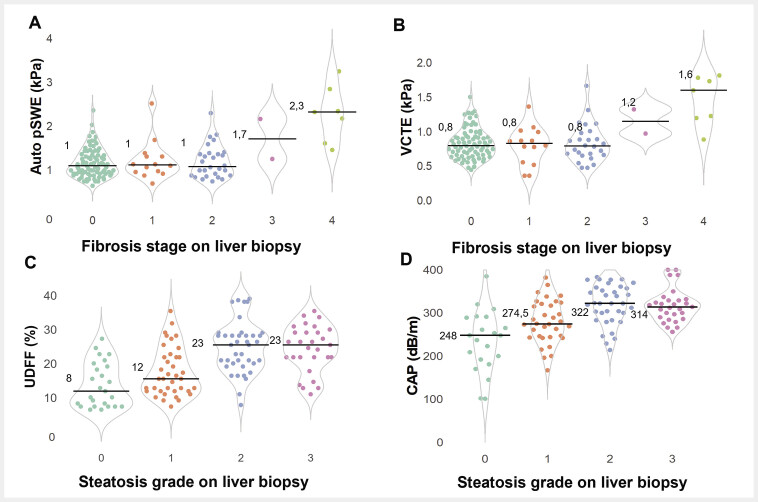
Beeswarm/violin plots of ( **A** ) log-transformed Auto pSWE and ( **B** ) log-transformed reliable VCTE according to histological fibrosis stage, and ( **C** ) UDFF and ( **D** ) reliable CAP according to histological steatosis grade. Individual measurements are represented as points corresponding to the stage of fibrosis or grade of steatosis, with median values indicated by a solid black line. Abbreviations: Auto pSWE: automated point shear wave elastography; CAP: controlled attenuation parameter; UDFF: ultrasound-derived fat fraction; VCTE: vibration-controlled transient elastography

**Fig. 3:**
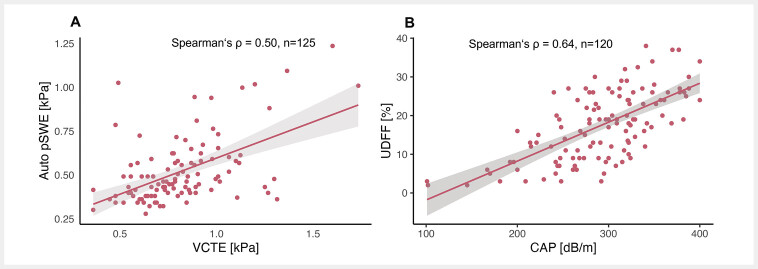
Scatter plots illustrating the correlation between ( **A** ) log-transformed reliable VCTE and log-transformed Auto pSWE and ( **B** ) reliable CAP and UDFF measurements. The Spearman correlation coefficients and the number of individuals included are displayed in the upper left corner. Abbreviations: Auto pSWE: automated point shear wave elastography; CAP: controlled attenuation parameter; UDFF: ultrasound-derived fat fraction; VCTE: vibration-controlled transient elastography

**Fig. 4:**
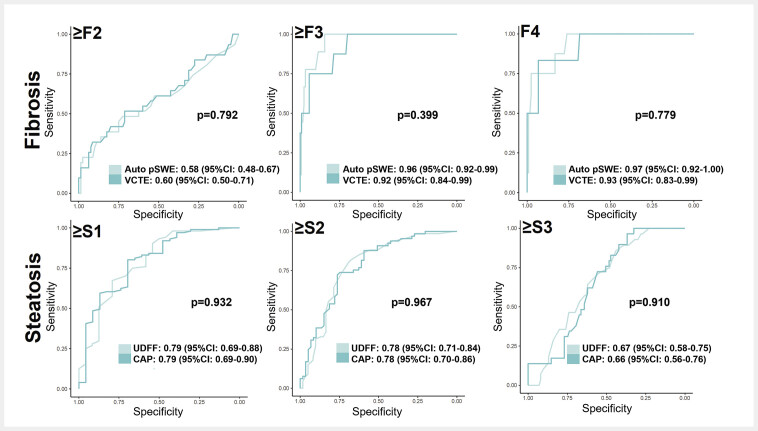
ROC curves for the detection of (≥F2) significant fibrosis, (≥F3) advanced fibrosis, (F4) cirrhosis for Auto pSWE and VCTE, as well as for the detection of (≥S1) mild steatosis, (≥S2) moderate steatosis, and (≥S3) severe steatosis for CAP and UDFF. The curves are presented with AUC with the corresponding 95% confidence intervals and p-values determined through bootstrapping (n=2000). Abbreviations: Auto pSWE: automated point shear wave elastography; CAP: controlled attenuation parameter; ROC: receiver operator characteristics; UDFF: ultrasound-derived fat fraction; VCTE: vibration-controlled transient elastography

When comparing noninvasive measurements across fibrosis stages ( Fig. 2 ), Auto pSWE-LSM produced results similar to VCTE-LSM for F0/1 and F2 but showed higher values for F3 and F4. A moderate correlation was observed between VCTE and Auto pSWE measurements (Spearman’s ρ=0.50, p<0.001; Fig. 3 ). No significant difference in diagnostic accuracy (AUC) was found between a single composite Auto pSWE acquisition (1×15 LSMs) and the median of 5 acquisitions (5×15 LSMs). Using a reliability criterion (IQR/Med ≤30%) for 5 Auto pSWE-LSMs did not improve the AUCs compared to single measurements ( Table 2 ). Consequently, single composite (1×15) Auto pSWE LSM results were used for further analyses. Auto pSWE demonstrated the highest diagnostic AUCs for advanced fibrosis (≥F3: AUC 0.96, CI 95%: 0.92–0.99) and cirrhosis (F4: AUC 0.97, CI 95%: 0.92–1.00). The diagnostic accuracy to detect significant fibrosis (≥F2) was poor, with an AUC of 0.58 (CI95%: 0.48–0.67, Table 3 ). No significant differences in diagnostic accuracy between VCTE and Auto pSWE were identified for any fibrosis stage ( Fig. 4 ). Cut-offs, sensitivities, specificities, and predictive values for Auto pSWE and VCTE for detecting significant fibrosis (≥F2), severe fibrosis (≥F3), and cirrhosis (F4) are summarized in Table 3 .

**Table:** **Table 2** Comparison of model characteristics/AUCs of median of measurements vs. single measurement with 95%-confidence intervals (in brackets) using a bootstrap test with n=2000 bootstraps for associated ROC curves. Power values for the comparisons of Auto pSWE vs. VCTE and UDFF vs. CAP are included in the right column.

	**Single Auto pSWE**	**Median of Five Auto pSWE acquisitions**	**p-value**	
Significant fibrosis (≥F2)	0.58 (0.46–0.70)	0.61 (0.50–0.73)	0.334	
Advanced fibrosis (≥F3)	0.96 (0.92–1.00)	0.92 (0.84–1.00)	0.170	
Cirrhosis (F4)	0.97 (0.92–1.00)	0.95 (0.90–1.00)	0.342	
	**Single Auto pSWE**	**VCTE**	**p-value**	**Power**
Significant fibrosis (≥F2)	0.58 (0.46–0.70)	0.61 (0.49–0.72)	0.648	0.113
Advanced fibrosis (≥F3)	0.96 (0.92–1.00)	0.91 (0.83–1.00)	0.305	0.162
Cirrhosis (F4)	0.97 (0.92–1.00)	0.92 (0.83–1.00)	0.397	0.113
	**Single UDFF**	**Median of Five UDFF acquisitions**	**p-value**	
Mild steatosis (≥S1)	0.79 (0.69–0.88)	0.79 (0.70–0.89)	0.986	
Moderate steatosis (≥S2)	0.78 (0.71–0.84)	0.80 (0.73–0.88)	0.381	
Severe steatosis (S3)	0.67 (0.58–0.75)	0.66 (0.57–0.76)	0.637	
	**Single UDFF**	**CAP**	**p-value**	**Power**
Mild steatosis (≥S1)	0.79 (0.69–0.88)	0.79 (0.69–0.90)	0.934	0.054
Moderate steatosis (≥S2)	0.78 (0.71–0.84)	0.78 (0.70–0.86)	0.967	0.051
Severe steatosis (S3)	0.67 (0.58–0.75)	0.66 (0.56–0.76)	0.908	0.054
Abbreviations: Auto pSWE: automated point shear wave elastography; CAP: controlled attenuation parameter; UDFF: ultrasound-derived fat fraction; VCTE: vibration-controlled transient elastography.				

**Table:** **Table 3** Diagnostic accuracy and optimal cut-offs for liver fibrosis stages of Auto pSWE and VCTE.

Elastography Technique	Stage	AUC (95%CI)		Cut-off	Sensitivity, % (95%CI)	Specificity, % (95%CI)	PPV, % (95%CI)	NPV, % (95%CI)	DLR +	DLR –	DOR
**Auto pSWE**	*Significant fibrosis (≥ F2)*	0.58 (0.48–0.67)	Youden	≥5.8 kPa	21.6 (9.8–38.2)	92.8 (85.7–97)	53.3 (26.6–78.7)	75.6 (66.9–83)	3	0.84	3.55
			Rule-in	≥6.0 kPa	21.6 (9.8–38.2)	95.9 (89.8–98.9)	66.7 (34.9–90.1)	76.2 (67.7–83.5)	5.24	0.82	6.41
			Rule-out	< 2.2 kPa	89.2 (74.6–97)	11.3 (5.8–19.4)	27.7 (19.9–36.7)	73.3 (5.8–19.4)	1.01	0.95	1.06
	*Advanced fibrosis (≥ F3)*	0.96 (0.92–0.99)	Youden	≥6.2 kPa	77.8 (40–97.2)	96 (90.9–98.7)	58.3 (27.7–84.8)	98.4 (90.9–98.7)	19.44	0.23	84
			Rule-in	≥6.7 kPa	77.8 (40–97.2)	96.8 (92–99.1)	63.6 (30.8–89.1)	98.4 (92–99.1)	24.31	0.23	105.88
			Rule-out	<4.7 kPa	100 (66.4–100)	84.8 (77.3–90.6)	32.1 (15.9–52.4)	100 (96.6–100)	6.58	0	Inf
	*Liver cirrhosis (F4)*	0.97 (0.92–1.00)	Youden	≥6.6 kPa	85.7 (42.1–99.6)	96.9 (92.1–99.1)	60 (26.2–87.8)	99.2 (95.6–100)	27.21	0.15	184.5
			Rule-in	≥ 7.9 kPa	85.7 (42.1–99.6)	97.6 (93.3–99.5)	66.7 (29.9–92.5)	99.2 (95.6–100)	36.29	0.15	248
			Rule-out	<4.7 kPa	100 (59–100)	83.5 (75.8–89.5)	25 (10.7–44.9)	100 (96.6–100)	6.05	0	Inf
**VCTE**	*Significant fibrosis (≥ F2)*	0.60 (0.50–0.71)	Youden	≥13.3 kPa	22.6 (9.6–41.1)	92.8 (84.9–97.3)	53.8 (25.1–80.8)	76.2 (66.7–84.1)	3.12	0.83	3.74
			Rule-in	≥12.6 kPa	32.3 (16.7–51.4)	89.2 (80.4–94.9)	52.6 (28.9–75.6)	77.9 (68.2–85.8)	2.97	0.76	3.92
			Rule-out	<3.5 kPa	90.3 (74.2–98)	7.2 (2.7–15.1)	26.7 (18.5–36.2)	66.7 (29.9–92.5)	0.97	1.34	0.73
	*Advanced fibrosis (≥ F3)*	0.92 (0.84–0.99)	Youden	≥15.0 kPa	75 (34.9–96.8)	94.3 (88.1–97.9)	50 (21.1–78.9)	98 (93.1–99.8)	13.25	0.26	50
			Rule-in	≥13.7 kPa	75 (34.9–96.8)	93.4 (86.9–97.3)	46.2 (19.2–74.9)	98 (93–99.8)	11.36	0.27	42.43
			Rule-out	<7.9 kPa	87.5 (47.3–99.7)	72.6 (63.1–80.9)	19.4 (8.2–36)	98.7 (93.1–100)	3.2	0.17	18.59
	*Liver cirrhosis (F4)*	0.93 (0.83–0.99)	Youden	≥15.8 kPa	66.7 (22.3–95.7)	93.5 (87.1–97.4)	36.4 (10.9–69.2)	98.1 (93.2–99.8)	10.29	0.36	28.86
			Rule-in	≥13.0 kPa	83.3 (35.9–99.6)	89.8 (82.5–94.8)	31.2 (11–58.7)	99 (94.4–100)	8.18	0.19	44.09
			Rule-out	<7.7 kPa	100 (54.1–100)	68.5 (58.9–77.1)	15 (5.7–29.8)	100 (95.1–100)	3.18	0	Inf
Abbreviations: 95%CI: 95%-confidence interval; AUC: area under the curve of the receiver operator characteristics; Auto pSWE: automated point shear wave elastography; DLR: diagnostic likelihood ratio; DOR: diagnostic odds ratio; NPV: negative predictive value; PPV: positive predictive value; VCTE: vibration-controlled transient elastography.											

### UDFF vs. VCTE-CAP for noninvasive steatosis assessment

UDFF and CAP steadily increased with histological steatosis grades but levelled off at moderate/severe steatosis ( Fig. 2 ). The AUC for a single UDFF acquisition did not differ significantly from the median of five acquisitions, so single measurements were used for all analyses ( Table 2 ). Reliable CAP and UDFF measurements showed a strong correlation (Spearman’s ρ=0.64, p<0.001; Fig. 3 ). UDFF demonstrated the highest diagnostic accuracy for mild (≥S1; AUC 0.79, CI 95%: 0.69–0.88) and moderate (≥S2; AUC 0.78, CI 95%: 0.71–0.84) steatosis but moderate accuracy for severe steatosis (S3; AUC 0.67, CI 95%: 0.58–0.75; Table 4 ). No significant differences in diagnostic accuracy between UDFF and CAP were found for any steatosis grade ( Fig. 4 ). Rule-in and rule-out cut-offs, along with sensitivity, specificity, and predictive values for UDFF and CAP, are detailed in Table 4 . Power calculations (bootstrapping, n = 2000) for VCTE vs. Auto pSWE and CAP vs. UDFF indicate that the low power is due to the small AUC differences, supporting the conclusion that these methods yield similar diagnostic performance ( Table 2 ).

**Table:** **Table 4** Diagnostic accuracy and optimal cut-offs for liver steatosis grades of UDFF and CAP.

Liver Fat Quantification Technique	Grade	AUC (95%CI)		Cut-off	Sensitivity, % (95%CI)	Specificity, % (95%CI)	PPV, % (95%CI)	NPV, % (95%CI)	DLR +	DLR –	DOR
**UDFF**	*Mild steatosis (≥ S1)*	0.79 (0.69–0.88)	Youden	≥16 %	67.3 (57.4–76.2)	79.2 (57.8–92.9)	93.3 (85.1–97.8)	35.8 (23.1–50.2)	3.23	0.41	7.82
			Rule-in	≥19 %	51 (41–60.9)	87.5 (67.6–97.3)	94.6 (85.1–98.9)	29.2 (19–41.1)	4.08	0.56	7.27
			Rule-out	<8 %	90.4 (83–95.3)	54.2 (32.8–74.4)	89.5 (82–94.7)	56.5 (34.5–76.8)	1.97	0.18	11.11
	*Moderate steatosis (≥ S2)*	0.78 (0.71–0.84)	Youden	≥17.1 %	73.1 (60.9–83.2)	73.8 (60.9–84.2)	75.4 (63.1–85.2)	71.4 (58.7–82.1)	2.79	0.36	7.66
			Rule-in	≥28 %	31.3 (20.6–43.8)	90.2 (79.8–96.3)	77.8 (57.7–91.4)	54.5 (44.2–64.4)	3.19	0.76	4.18
			Rule-out	<12 %	91 (81.5–96.6)	45.9 (33.1–59.2)	64.9 (54.4–74.5)	82.4 (65.5–93.2)	1.68	0.2	8.63
	*Severe steatosis (S3)*	0.67 (0.58–0.75)	Youden	≥16 %	75 (55.1–89.3)	48 (37.9–58.2)	28.8 (18.8–40.6)	87.3 (75.5–94.7)	1.44	0.52	2.77
			Rule-in	≥32 %	10.7 (2.3–28.2)	90 (82.4–95.1)	23.1 (5–53.8)	78.3 (69.6–85.4)	1.07	0.99	1.08
			Rule-out	<14 %	89.3 (71.8–97.7)	39 (29.4–49.3)	29.1 (19.8–39.9)	92.9 (80.5–98.5)	1.46	0.27	5.33
**CAP**	*Mild steatosis (≥ S1)*	0.79 (0.69–0.90)	Youden	≥265.5 dB/m	81.7 (70.7–89.9)	71.4 (41.9–91.6)	93.5 (84.3–98.2)	43.5 (23.2–65.5)	2.86	0.26	11.15
			Rule-in	≥310 dB/m	51.0 (41.0–60.9)	87.5 (67.6–97.3)	94.6 (85.1–98.9)	29.2 (19.0–41.1)	4.08	0.56	7.27
			Rule-out	<242 dB/m	90.4 (83.0–95.3)	54.2 (32.8–74.4)	89.5 (82.0–94.7)	56.5 (34.5–76.8)	1.97	0.18	11.11
	*Moderate steatosis (≥ S2)*	0.78 (0.70–0.86)	Youden	≥298.5 dB/m	78.7 (64.3–89.3)	60.5 (43.4–76)	71.2 (56.9–82.9)	69.7 (51.3–84.4)	1.99	0.35	5.67
			Rule-in	≥339 dB/m	31.3 (20.6–43.8)	90.2 (79.8–96.3)	77.8 (57.7–91.4)	54.5 (44.2–64.4)	3.19	0.76	4.18
			Rule-out	<266 dB/m	91 (81.5–96.6)	45.9 (33.1–59.2)	64.9 (54.4–74.5)	82.4 (65.5–93.2)	1.68	0.20	8.63
	*Severe steatosis (S3)*	0.66 (0.56–0.76)	Youden	≥265.5 dB/m	90 (68.3–98.8)	35.4 (23.9–48.2)	30 (18.8–43.2)	92 (74.0–99.0)	1.39	0.28	4.93
			Rule-in	≥366 dB/m	10.7 (2.3–28.2)	90 (82.4–95.1)	23.1 (5–53.8)	78.3 (69.6–85.4)	1.07	0.99	1.08
			Rule-out	<266 dB/m	89.3 (71.8–97.7)	39 (29.4–49.3)	29.1 (19.8–39.9)	92.9 (80.5–98.5)	1.46	0.27	5.33
Abbreviations: 95%CI: 95%-confidence interval; AUC: area under the curve of the receiver operator characteristics; CAP: controlled attenuation parameter; DLR: diagnostic likelihood ratio; DOR: diagnostic odds ratio; UDFF: ultrasound-derived fat fraction; NPV: negative predictive value; PPV: positive predictive value.											

## Discussion

VCTE-LSM and CAP are established tools for noninvasive fibrosis and steatosis assessment but require a specialized and costly device and do not allow the acquisition of regular B-mode ultrasound images. While it is recommended to obtain ≥10 reliable measurements for VCTE-based assessments 16 , the automated measurement sequence of Auto pSWE (15 single LSMs) and UDFF takes only eight seconds (including cooling time) in addition to a routine liver ultrasound examination. However, the total procedure time varies depending on factors such as patient positioning, acoustic window optimization, and image quality adjustment – challenges that can be particularly pronounced in obese patients. This study evaluated the diagnostic accuracy of Auto pSWE and UDFF compared to the LSM and CAP obtained by VCTE using liver biopsy as a reference standard in predominantly obese MASLD patients. While liver biopsy remains the gold standard, its invasive nature poses risks and limits its routine use 17 , highlighting the need for accessible, repeatable, and accurate noninvasive assessment tools.

A single Auto pSWE/UDFF measurement proved to be as accurate as the median of five measurements for diagnosing fibrosis and steatosis. Additionally, applying a quality criterion of IQR/Med ≤ 30% to five Auto pSWE LSMs did not enhance diagnostic accuracy, indicating that a solitary measurement is adequate for risk assessment. However, potential reliability criteria warrant future investigation.

Auto pSWE-LSM showed the highest diagnostic accuracy for advanced fibrosis (≥F3) and cirrhosis (F4) with AUCs of 0.96 and 0.97, respectively, thereby marginally outperforming VCTE. Both methods meet the criterion recommended by the European Association for the Study of the Liver (EASL) for noninvasive fibrosis assessment 17 . However, similar cut-offs between fibrosis stages limit precise staging capabilities, suggesting their primary value lies in identifying significant fibrosis risk rather than exact staging.

UDFF and CAP showed comparable diagnostic accuracy for all grades of steatosis, performing best with regard to detecting any steatosis (≥S1) but less effectively with respect to grading severity. The close cut-off values between steatosis grades suggest that these tools are more suitable for detecting the presence of steatosis rather than grading steatosis, particularly in patients with severe obesity.

The limited accuracy of CAP for reliably differentiating advanced steatosis grades has been previously highlighted 18 19 . This limitation is particularly relevant in patients with severe obesity, where acoustic interference can further reduce the diagnostic accuracy of CAP. Despite these limitations, assessing steatosis remains clinically important, especially for longitudinal monitoring of patients undergoing bariatric surgery, where changes in hepatic fat content can indicate therapeutic effects 20 21 . UDFF and Auto pSWE measure different aspects of liver pathology: UDFF quantifies hepatic fat content, while Auto pSWE assesses liver stiffness linked to fibrosis. As such, these methods serve as complementary tools in the noninvasive assessment of liver disease.

This study has limitations: First, while steatosis grades are well distributed, the population included few patients with ≥F3, introducing a risk of spectrum bias and potentially overestimating the diagnostic accuracy of Auto pSWE and VCTE for detecting advanced fibrosis (F3/F4), as a small sample size of higher fibrosis stages can inflate AUC values. While further studies with a more even distribution of the fibrosis spectrum are warranted, it is important to note that our cohort is representative of a real-world MASLD population with severe obesity, where the prevalence of advanced fibrosis is inherently lower. Moreover, four patients underwent transjugular liver biopsy. While transjugular biopsies are more common in individuals with (suspected) higher fibrosis stages, in our cohort, only two of four transjugular liver biopsies showed F4 fibrosis, limiting a potential impact on our findings. Second, liver biopsy variability complicates its role as a reference standard. Histological accuracy hinges on the quality of the biopsy, i.e., the length of the biopsy and the number of portal fields examined. Shorter biopsies and fewer portal tracts risk misrepresenting fibrosis severity. In our study, the median biopsy length (1.9 cm) and the median number of portal fields 8 were slightly below the recommended thresholds (≥2.0 cm and ≥11 portal tracts), reflecting routine practice but introducing a margin of error 22 . Third, with the new definition of MASLD, the clinical spectrum of steatotic liver disease includes varying etiologies (e.g., ArLD, MetALD), necessitating further evaluation of Auto pSWE and UDFF across diverse populations 23 . Our study cohort mainly included patients with metabolic liver disease, warranting further studies on the diagnostic accuracy of Auto pSWE and UDFF in diverse populations and etiologies 24 . Additionally, future research should assess the accuracy of Auto pSWE for detecting clinically significant portal hypertension (CSPH) 25 , which may be a relevant predictor of morbidity and mortality in MASLD 26 .

Fourth, the cut-off values derived in this study reflect our cohort’s fibrosis prevalence and likely require adjustment based on pre-test probability in different target populations. Further validation in diverse patient cohorts is warranted to ensure broader clinical applicability. Lastly, while Auto pSWE and UDFF are accurate for detecting fibrosis and steatosis, caution is needed for detailed staging or grading. These tools are best used as part of broader clinical assessments with rule-in and rule-out cut-offs guiding management, without replacing more precise diagnostic tools when detailed grading is required.

In summary, our study shows that a single Auto pSWE acquisition (15 composite LSMs) and UDFF represent accurate, noninvasive tools for assessing advanced fibrosis and steatosis in obese MASLD patients. This time-efficient approach achieves diagnostic accuracy comparable to VCTE-based LSM and CAP and can be seamlessly integrated into routine ultrasound examinations for precise liver steatosis quantification and fibrosis risk assessment.

## Supplementary Material

Supplementary Material Zusatzmaterial

## References

[1] Riazi K Azhari H Charette JH The prevalence and incidence of NAFLD worldwide: a systematic review and meta-analysis Lancet Gastroenterol Hepatol 2022 7 09 851 861 10.1016/S2468-1253(22)00165-0 35798021

[2] Loomba R Wong R Fraysse J Nonalcoholic fatty liver disease progression rates to cirrhosis and progression of cirrhosis to decompensation and mortality: a real world analysis of Medicare data Aliment Pharmacol Ther 2020 51 11 1149 1159 10.1111/apt.15679 32372515

[3] Castera L Friedrich-Rust M Loomba R Noninvasive Assessment of Liver Disease in Patients With Nonalcoholic Fatty Liver Disease Gastroenterology 2019 156 05 1264 1281 10.1053/j.gastro.2018.12.036 30660725 PMC7505052

[4] Semmler G Wöran K Scheiner B Novel reliability criteria for controlled attenuation parameter assessments for non-invasive evaluation of hepatic steatosis United European Gastroenterol J 2020 8 03 321 331 10.1177/2050640619900820 PMC7184665 32213023

[5] Kubale R Schneider G Lessenich CPN Ultrasound-Derived Fat Fraction for Hepatic Steatosis Assessment: Prospective Study of Agreement With MRI PDFF and Sources of Variability in a Heterogeneous Population AJR Am J Roentgenol 2024 222 06 e2330775 38506537 10.2214/AJR.23.30775

[6] Wan T Köhn N Kröll D Applicability and Results of Liver Stiffness Measurement and Controlled Attenuation Parameter Using XL Probe for Metabolic-Associated Fatty Liver Disease in Candidates to Bariatric Surgery. A Single-Center Observational Study Obes Surg 2021 31 02 702 711 10.1007/s11695-020-04971-w 32959331

[7] Wong VW Irles M Wong GL Unified interpretation of liver stiffness measurement by M and XL probes in non-alcoholic fatty liver disease Gut 2019 68 11 2057 2064 10.1136/gutjnl-2018-317334 30658997

[8] Gao J Wong C Maar M Reliability of performing ultrasound derived SWE and fat fraction in adult livers Clin Imaging 2021 80 424 429 10.1016/j.clinimag.2021.08.025 34543866

[9] Barr RG Wilson SR Rubens D Update to the Society of Radiologists in Ultrasound Liver Elastography Consensus Statement Radiology 2020 296 02 263 274 10.1148/radiol.2020192437 32515681

[10] Blank V Petroff D Wiegand J M probe comes first: Impact of initial probe choice on diagnostic performance of vibration controlled transient elastography Dig Liver Dis 2022 54 03 358 364 10.1016/j.dld.2021.08.003 34446354

[11] Schwabl P Bota S Salzl P New reliability criteria for transient elastography increase the number of accurate measurements for screening of cirrhosis and portal hypertension Liver Int 2015 35 02 381 390 10.1111/liv.12623 24953516

[12] Reiberger T Schwabl P Trauner M Measurement of the Hepatic Venous Pressure Gradient and Transjugular Liver Biopsy J Vis Exp 2020 160 10.3791/58819 32628153

[13] Vienna MUo Laboratory reference values: AKH Wien; 2024. Available from: https://www.akhwien.at/default.aspx?pid=3985

[14] Hinkle DE Wiersma W Jurs SG Applied statistics for the behavioral sciences Boston Houghton Mifflin 2003 756

[15] Hosmer DW Lemeshow S Sturdivant RX Applied logistic regression Hoboken, New Jersey Wiley 2013

[16] Bauer DJM Nixdorf L Dominik N The deep abdominal ultrasound transducer (DAX) increases the success rate and diagnostic accuracy of shear wave elastography for liver fibrosis assessment in patients with obesity-A prospective biopsy-controlled study Aliment Pharmacol Ther 2024 60 01 70 82 10.1111/apt.18019 38693718

[17] EASL Clinical Practice Guidelines on non-invasive tests for evaluation of liver disease severity and prognosis – 2021 update J Hepatol 2021 75 03 659 689 10.1016/j.jhep.2021.05.025 34166721

[18] Newsome PN Sasso M Deeks JJ FibroScan-AST (FAST) score for the non-invasive identification of patients with non-alcoholic steatohepatitis with significant activity and fibrosis: a prospective derivation and global validation study Lancet Gastroenterol Hepatol 2020 5 04 362 373 10.1016/S2468-1253(19)30383-8 32027858 PMC7066580

[19] Petroff D Blank V Newsome PN Assessment of hepatic steatosis by controlled attenuation parameter using the M and XL probes: an individual patient data meta-analysis Lancet Gastroenterol Hepatol 2021 6 03 185 198 10.1016/S2468-1253(20)30357-5 33460567

[20] Karlas T Petroff D Feisthammel J Endoscopic Bariatric Treatment with Duodenal-Jejunal Bypass Liner Improves Non-invasive Markers of Non-alcoholic Steatohepatitis Obes Surg 2022 32 08 2495 2503 10.1007/s11695-022-06150-5 35713854 PMC9273553

[21] Nixdorf L Hartl L Ströhl S Rapid improvement of hepatic steatosis and liver stiffness after metabolic/bariatric surgery: a prospective study Sci Rep 2024 14 01 17558 10.1038/s41598-024-67415-w 39080285 PMC11289378

[22] Neuberger J Patel J Caldwell H Guidelines on the use of liver biopsy in clinical practice from the British Society of Gastroenterology, the Royal College of Radiologists and the Royal College of Pathology Gut 2020 69 08 1382 1403 10.1136/gutjnl-2020-321299 32467090 PMC7398479

[23] Rinella ME Lazarus JV Ratziu V A multisociety Delphi consensus statement on new fatty liver disease nomenclature J Hepatol 2023 79 06 1542 1556 10.1097/HEP.0000000000000520 37364790

[24] Bauer DJM Silvestri IA Mare R Two-dimensional shear wave elastography (ElastQ) accurately rules out liver fibrosis and rules in advanced chronic liver disease across liver disease etiologies: a prospective multicenter study Ultrasonography 2023 42 04 544 554 10.1002/sono.12416 37644806 PMC10555684

[25] Reiberger T The Value of Liver and Spleen Stiffness for Evaluation of Portal Hypertension in Compensated Cirrhosis Hepatol Commun 2022 6 05 950 964 10.1002/hep4.1855 34904404 PMC9035575

[26] Mitten EK Portincasa P Baffy G Portal Hypertension in Nonalcoholic Fatty Liver Disease: Challenges and Paradigms J Clin Transl Hepatol 2023 11 05 1201 1211 10.14218/JCTH.2023.00029 37577237 PMC10412712

