# Supplementary material for: UDFF and Auto pSWE accurately assess liver steatosis and fibrosis risk in obese patients with MASLD
Source: Ultraschall Med. 2025 Aug 7;46(6):603–13. doi: 10.1055/a-2592-1431 (PMC12700709; doi:10.1055/a-2592-1431)
Supplement: Supplementary file 1 — Supplementary Material [file 10-1055-a-2592-1431_25942817.pdf]

Ultraschall in Med | 10.1055/a-2592-1431 | © The Author(s).

SUPPLEMENT

Supplementary Table 1: Detailed results of liver biopsy categorized by fibrosis stage

	Overall, n=134	No Fibrosis, n=83	Mild Fibrosis, n=14	Significant Fibrosis, n=28	Advanced Fibrosis, n=2	Liver Cirrhosis, n=7	p-value
**Histological Activity Grade, n (%)**							
0	59 (44.0%)	49 (59.0%)	6 (42.9%)	3 (10.7%)	0 (0%)	1 (14.3%)	**<0.001**
1	69 (51.5%)	34 (41.0%)	7 (50.0%)	21 (75.0%)	2 (100%)	5 (71.4%)	
2	5 (3.7%)	0 (0%)	1 (7.1%)	4 (14.3%)	0 (0%)	0 (0%)	
**Histological Steatosis Grade, n (%)**							
0	25 (18.7%)	20 (24.1%)	2 (14.3%)	0 (0%)	1 (50.0%)	2 (28.6%)	0.510
1	38 (28.4%)	21 (25.3%)	4 (28.6%)	10 (35.7%)	1 (50.0%)	2 (28.6%)	
2	41 (30.6%)	27 (32.5%)	5 (35.7%)	8 (28.6%)	0 (0%)	1 (14.3%)	
3	30 (22.4%)	15 (18.1%)	3 (21.4%)	10 (35.7%)	0 (0%)	2 (28.6%)	

Abbreviations: n: number

Thieme

Ultraschall in Med | 10.1055/a-2592-1431 | © The Author(s).

Supplementary Table 2: Population characteristics by fibrosis stage from liver biopsy. The table compares characteristics, with statistical significance assessed by Chi-squared or Kruskal-Wallis test as appropriate, indicated by p-values.

**Histological Fibrosis Stage**	**Overall, n=134**	**No Fibrosis, n=83**	**Mild Fibrosis, n=14**	**Significant Fibrosis, n=28**	**Advanced Fibrosis, n=2**	**Liver Cirrhosis, n=7**	**p-value**
**Age, years, mean ± SD**	42.6 ± 13.3	41.5 ± 12.7	44.6 ± 16.2	40.5 ± 11.0	63.0 ± 9.90	54.9 ± 14.6	0.070
**Sex, n (%)**							
Male	47 (35.1%)	22 (26.5%)	8 (57.1%)	10 (35.7%)	1 (50.0%)	6 (85.7%)	**0.017**
Female	87 (64.9%)	61 (73.5%)	6 (42.9%)	18 (64.3%)	1 (50.0%)	1 (14.3%)	
**BMI, kg/m², mean ± SD**	42.7 ± 10.4	44.0 ± 9.7	42.4 ± 11.7	44.0 ± 9.4	24.8 ± 7.5	28.0 ± 5.2	**0.002**
**Platelets, G/L, median [IQR]**	249 [106]	258 [105]	244 [34.0]	254 [83.3]	NA	115 [107]	**0.012**
**INR (% of ULN), median [IQR]**	96 [17]	96 [9]	96 [17]	87 [11]	96 [0]	130 [17]	**0.001**
**Bilirubin (% of ULN), median [IQR]**	157 [106]	147 [86]	169 [102]	167 [88]	157 [44]	427 [220]	**0.022**
**Albumin, g/dL, median [IQR]**	40.9 [5.1]	40.9 [3.5]	39.6 [5.8]	41.8 [6.9]	NA	38.1 [9.3]	0.432
**MELD-Na, points, median [IQR]**	7 [2]	7 [2]	8 [3]	7 [2]	NA	10 [1]	**0.017**
**ALT (% of ULN), median [IQR]**	94 [78]	92 [80]	94 [108]	111 [86]	173 [47]	82 [33]	**0.246**
**AST (% of ULN), median [IQR]**	69 [35]	66 [38]	66 [30]	80 [30]	133 [5]	84 [84]	**0.030**
**ALP (% of ULN), median [IQR]**	65 [28]	65 [30]	54 [23]	66 [18]	75 [13]	97 [13]	0.124
**GGT (% of ULN), median [IQR]**	160 [75]	159 [79]	127 [70]	171 [55]	181 [48]	210 [43]	0.231
**HbA1c, %, median [IQR]**	5.5 [1.4]	5.3 [1.0]	6.2 [1.8]	5.5 [1.4]	NA	5.1 [1.2]	0.279
**ELF test, points, median [IQR]**	8.9 [1.9]	8.8 [1.5]	8.6 [2.1]	8.3 [1.8]	NA	11.4 [2.0]	**0.009**
**Etiology, n (%)**							
MASLD/MASH	118 (88.1%)	77 (92.8%)	11 (78.6%)	26 (92.9%)	1 (50.0%)	3 (42.9%)	**<0.001**
ArLD	3 (2.2%)	1 (1.2%)	0 (0%)	0 (0%)	1 (50.0%)	1 (14.3%)	
Viral	2 (1.5%)	0 (0%)	0 (0%)	1 (3.6%)	0 (0%)	1 (14.3%)	
Others	11 (8.2%)	5 (6.0%)	3 (21.4%)	1 (3.6%)	0 (0%)	2 (28.6%)	
**VCTE probe, n (%)**							
M	35 (26.1%)	14 (16.9%)	6 (42.9%)	8 (28.6%)	0 (0%)	7 (100%)	**0.002**

Thieme

Histological Fibrosis Stage	Overall, n=134	No Fibrosis, n=83	Mild Fibrosis, n=14	Significant Fibrosis, n=28	Advanced Fibrosis, n=2	Liver Cirrhosis, n=7	p-value
XL	92 (68.7%)	64 (77.1%)	8 (57.1%)	18 (64.3%)	2 (100%)	0 (0%)	
Failed	7 (5.2%)	5 (6.0%)	0 (0%)	2 (7.1%)	0 (0%)	0 (0%)	
**VCTE, kPa, median [IQR]**	6.6 [5.3]	6.2 [3.9]	6.8 [5.1]	6.2 [4.5]	NA	39.9 [41.1]	**0.004**
**DAX-Auto pSWE median of 5 measurements, kPa, median [IQR]**	3.1 [1.6]	3.0 [1.3]	3.1 [1.1]	3.0 [1.5]	NA	10.2 [6.9]	**0.003**
**DAX-Auto pSWE – single measurement, kPa, median [IQR]**	3.0 [2.0]	2.9 [1.7]	3.1 [1.3]	2.8 [1.7]	NA	10.0 [2.6]	**<0.001**
**UDFF median of 5 measurements, %, median [IQR]**	18 [16]	19 [14]	14 [16]	19 [13]	NA	7 [11]	0.155
**UDFF median – single measurement, %, median [IQR]**	18 [14]	18 [17]	19 [17]	19 [13]	NA	12 [12]	0.273
**CAP, dB/m, median [IQR]**	295 [78]	293 [74]	319 [157]	315 [72]	NA	265 [0]	0.894
**cCAP, dB/m, median [IQR]**	300 [69]	300 [86]	268 [50]	300 [43]	NA	308 [25]	0.770

Abbreviations: ALT: alanine aminotransferase; ALP: alkaline phosphatase; ArLD: alcohol-related liver disease; AST: aspartate aminotransferase; Auto pSWE: automated point shear wave elastography; BMI: body mass index; (c)CAP: (continuous) controlled attenuation parameter; DAX: deep abdominal (transducer); ELF: enhanced liver fibrosis test; GGT: gamma-glutamyl transferase; HbA1c: glycated hemoglobin; INR: international normalized ratio; IQR: interquartile range; MASLD: metabolic dysfunction-associated liver disease; MELD-Na: model for end-stage liver disease sodium score; n: number; SD: standard deviation; UDFF: ultrasound-derived fat fraction; ULN: upper limit of normal; VCTE: vibration-controlled transient elastography

Supplementary Table 3: Quality metrics of liver biopsies of the overall cohort

**Variable**	**Median (IQR)**	**Minimum**	**Maximum**
**Biopsy length, cm**	1.9 (1.5-2.2)	1	8
**Number of portal fields, n**	8.0 (6.0-13.0)	1	27

Abbreviations: cm: centimeters, IQR: interquartile range; n: number

Supplementary Table 4: Detailed liver biopsy results categorized by steatosis grade

	Overall, n=134	No Steatosis, n=25	Mild Steatosis, n=38	Moderate Steatosis, n=41	Severe Steatosis, n=30	p-value
**Histological Activity Grade**						
0	59 (44.0%)	21 (84.0%)	18 (47.4%)	14 (34.1%)	6 (20.0%)	**<0.001**
1	69 (51.5%)	4 (16.0%)	18 (47.4%)	26 (63.4%)	21 (70.0%)	
2	5 (3.7%)	0 (0%)	1 (2.6%)	1 (2.4%)	3 (10.0%)	
**Histological Ballooning**						
0	40 (29.9%)	10 (40.0%)	16 (42.1%)	10 (24.4%)	4 (13.3%)	**0.028**
1	16 (11.9%)	0 (0%)	3 (7.9%)	5 (12.2%)	8 (26.7%)	
2	1 (0.7%)	0 (0%)	0 (0%)	0 (0%)	1 (3.3%)	
3	40 (29.9%)	10 (40.0%)	16 (42.1%)	10 (24.4%)	4 (13.3%)	
**NAS Score, points, median [IQR]**	2 [2.5]	0 [0]	1.5 [1]	3 [0]	5 [1]	**<0.001**

Abbreviations: n: number; IQR: interquartile range

Ultraschall in Med | 10.1055/a-2592-1431 | © The Author(s).

Thieme

Ultraschall in Med | 10.1055/a-2592-1431 | © The Author(s).

Supplementary Table 5: Population characteristics by steatosis grade from liver biopsy. The table compares characteristics, with statistical significance assessed by Chi-squared or Kruskal-Wallis test as appropriate, indicated by p-values.

**Histological Steatosis Grade**	**Overall, n=134**	**No Steatosis, n=25**	**Mild Steatosis, n=38**	**Moderate Steatosis, n=41**	**Severe Steatosis, n=30**	**p-value**
**Age, years, mean ± SD**	42.6 ± 13.3	43.8 ± 16.0	43.3 ± 15.0	43.4 ± 11.7	39.7 ± 10.5	0.819
**Sex, n (%)**						
Male	47 (35.1%)	10 (40.0%)	9 (23.7%)	12 (29.3%)	16 (53.3%)	0.115
Female	87 (64.9%)	15 (60.0%)	29 (76.3%)	29 (70.7%)	14 (46.7%)	
**BMI, kg/m², mean ± SD**	42.7 ± 10.4	36.3 ± 12.2	42.3 ± 9.5	45.4 ± 8.8	44.9 ± 10.1	**0.036**
**Platelets, G/L, median [IQR]**	249 [106]	225 [168]	233 [72]	249 [97]	263 [103]	0.063
**INR (% of ULN), median [IQR]**	96 [17]	96 [10]	96 [17]	96 [17]	87 [9]	0.125
**Bilirubin (% of ULN), median [IQR]**	157 [106]	167 [147]	157 [119]	157 [60.0]	153 [90.5]	0.903
**Albumin, g/dL, median [IQR]**	40.9 [5.1]	40.8 [5.3]	41.1 [5.1]	40.2 [4.3]	41.6 [5.2]	0.607
**MELD-Na, points, median [IQR]**	7 [2]	8 [2]	7 [2]	7 [2]	6 [1]	0.001
**ALT (% of ULN), median [IQR]**	94 [78]	69 [58]	88 [54]	94 [72]	142 [89]	0.007
**AST (% of ULN), median [IQR]**	69[35]	63 [51]	63 [29]	70 [33]	80 [30]	0.659
**ALP (% of ULN), median [IQR]**	65 [28]	62 [31]	65 [27]	68 [31]	58 [21]	0.669
**GGT (% of ULN), median [IQR]**	160 [75]	159 [62]	163 [64]	178 [74]	138 [71]	0.471
**HbA1c, %, median [IQR]**	5.5 [1.4]	5.2 [0.4]	5.5 [1.8]	5.6 [1.8]	5.7 [1.1]	**0.045**
**ELF Test, points, median [IQR]**	8.9 [1.9]	8.9 [2.0]	8.9 [2.3]	8.9 [1.3]	8.7 [1.2]	0.976
**Etiology, n (%)**						
MASLD/MASH	118 (88.1%)	15 (60.0%)	34 (89.5%)	40 (97.6%)	29 (96.7%)	**0.022**
ArLD	3 (2.2%)	2 (8.0%)	0 (0%)	0 (0%)	1 (3.3%)	
Viral	2 (1.5%)	1 (4.0%)	0 (0%)	1 (2.4%)	0 (0%)	
Others	11 (8.2%)	7 (28.0%)	4 (10.5%)	0 (0%)	0 (0%)	
**VCTE probe, n (%)**						
M	35 (26.1%)	12 (48.0%)	11 (28.9%)	6 (14.6%)	6 (20.0%)	0.169

Thieme

Histological Steatosis Grade	Overall, n=134	No Steatosis, n=25	Mild Steatosis, n=38	Moderate Steatosis, n=41	Severe Steatosis, n=30	p-value
XL	92 (68.7%)	11 (44.0%)	26 (68.4%)	32 (78.0%)	23 (76.7%)	
Failed	7 (5.2%)	2 (8.0%)	1 (2.6%)	3 (7.3%)	1 (3.3%)	
**VCTE, kPa, median [IQR]**	6.6 [5.3]	6.9 [5.2]	6.1 [4.7]	6.3 [4.8]	7.4 [7.8]	0.697
**DAX-Auto pSWE median of 5 measurements, kPa, median [IQR]**	3.0 [2.0]	4.2 [3.0]	2.9 [2.6]	2.8 [1.3]	3.0 [1.6]	0.168
**DAX-Auto pSWE – single measurement, kPa, median [IQR]**	3.0 [2.0]	4.2 [3.0]	2.9 [2.6]	2.8 [1.3]	3.0 [1.6]	0.168
**UDFF median of 5 measurements, %, median [IQR]**	18 [16]	8 [13]	12 [11]	23 [9]	23 [8]	**<0.001**
**UDFF median – single measurement, %, median [IQR]**	18 [14]	7 [11]	14 [13]	23 [11]	22 [12]	**<0.001**
**CAP, dB/m, median [IQR]**	295 [78]	248 [118]	273 [71]	322 [70]	322 [87]	**0.033**
**cCAP, dB/m, median [IQR]**	300 [69]	243 [88]	278 [75]	339 [62]	313 [44]	**<0.001**

Abbreviations: ALT: alanine aminotransferase; ALP: alkaline phosphatase; ArLD: alcohol-related liver disease; AST: aspartate aminotransferase; Auto pSWE: automated point shear wave elastography; BMI: body mass index; (c)CAP: (continuous) controlled attenuation parameter; DAX: deep abdominal (transducer); ELF: enhanced liver fibrosis test; GGT: gamma-glutamyl transferase; HbA1c: glycated hemoglobin; INR: international normalized ratio; IQR: interquartile range; MASLD: metabolic dysfunction-associated liver disease; MELD-Na: model for end-stage liver disease sodium score; n: number; SD: standard deviation; UDFF: ultrasound-derived fat fraction; ULN: upper limit of normal; VCTE: vibration-controlled transient elastography

Ultraschall in Med | 10.1055/a-2592-1431 | © The Author(s).

Supplementary Material

Thieme

Supplementary Methods

For data management, Microsoft Excel was employed. Microsoft Word facilitated document creation and editing. GPT-4 was utilized exclusively for enhancing the text's style and readability. Statistical analyses were supported by specific R packages: cutpointr (1.1.2), ggplot2 (3.4.2), openxlsx (4.2.5.2), pROC (1.18.5), table1 (1.4.3), and tidyverse (2.0.0).
